# Ability to Work among Patients with ESKD: Relevance of Quality Care Metrics

**DOI:** 10.3390/healthcare5030042

**Published:** 2017-08-07

**Authors:** Nancy G. Kutner, Rebecca Zhang

**Affiliations:** 1 Department of Rehabilitation Medicine, Emory University School of Medicine, Atlanta, GA 30322, USA; 2 Department of Biostatistics and Bioinformatics, Rollins School of Public Health, Emory University, Atlanta, GA 30322, USA; hzhang3@emory.edu

**Keywords:** ability to work, dialysis, employment, end-stage kidney disease, quality indicators, quality of care

## Abstract

Enabling patient ability to work was a key rationale for enacting the United States (US) Medicare program that provides financial entitlement to renal replacement therapy for persons with end-stage kidney disease (ESKD). However, fewer than half of working-age individuals in the US report the ability to work after starting maintenance hemodialysis (HD). Quality improvement is a well-established objective in oversight of the dialysis program, but a more patient-centered quality assessment approach is increasingly advocated. The ESKD Quality Incentive Program (QIP) initiated in 2012 emphasizes clinical performance indicators, but a newly-added measure requires the monitoring of patient depression—an issue that is important for work ability and employment. We investigated depression scores and four dialysis-specific QIP measures in relation to work ability reported by a multi-clinic cohort of 528 working-age maintenance HD patients. The prevalence of elevated depression scores was substantially higher among patients who said they were not able to work, while only one of the four dialysis-specific clinical measures differed for patients able/not able to work. Ability to work may be among patients’ top priorities. As the parameters of quality assessment continue to evolve, increased attention to patient priorities might facilitate work ability and employment outcomes.

## 1. Introduction

In a recent international study, patients with end-stage kidney disease (ESKD) who were receiving hemodialysis (HD) named ability to work as one of their top ten priority outcomes [1]. Ability to sustain employment is associated not only with economic benefits, but also with benefits for an individual’s sense of self-worth and maintenance of social relationships. However, employment is a life area that may be dramatically disrupted for individuals with kidney disease who require ongoing renal replacement therapy (RRT), especially those who receive maintenance dialysis. Most working-age persons with ESKD who initiate maintenance dialysis treatment were employed before the start of treatment [2], but fewer than half of these persons report being “able” to work after RRT is initiated [3,4,5]. Dialysis clinics in the United States (US) are required to regularly report summary data documenting the facility’s performance on a series of dialysis quality of care clinical measures, but a more patient-centered approach to quality assessment that includes patient preferences is increasingly advocated [6]. We examined five currently monitored quality measures in relation to ability to work reported by a large US cohort of working-age individuals who were receiving clinic-based maintenance HD. We hypothesized that the proportion of patients meeting each quality of care target would be larger among those who reported ability to work than among those who did not report being able to work. 

### 1.1. Prevalent ESKD Patients in the US

Survival of persons with kidney disease who progress to kidney failure, ESKD, requires ongoing dialysis treatment or kidney transplantation. The most recent national data supplied by the US registry of persons with ESKD indicated that about 428,000 persons were receiving maintenance HD in calendar year 2014; about 47,500 used peritoneal dialysis; and about 200,000 had a functioning kidney transplant. Over half (53%) of prevalent HD patients were adults aged 22–64. The majority of persons on HD (88%) received their treatment “in-center,” typically in an outpatient clinic [7].

### 1.2. Work Ability and Employment among Persons with ESKD

An important rationale in 1972 for establishing the Medicare entitlement that funds treatment of ESKD in the US was the expectation that this program would enable most persons who required dialysis to return to work, because treatment would become more widely available and affordable. In the years following the establishment of the kidney disease Medicare program, increasing numbers of older and sicker individuals were accepted for dialysis as more facilities opened, resulting in a treated ESKD population for whom return to work was less feasible than was originally anticipated [8]. However, despite these demographic changes in the overall treated population, identifying strategies to promote employment opportunity among working-age persons on dialysis remains a valued objective [9,10,11,12].

In a 2000 Evidence Report/Technology Assessment, the US Agency for Healthcare Research and Quality attempted to specify the meaning of work ability/disability among persons with ESKD. The report addressed the question of whether the diagnosis of ESKD by itself is an appropriate criterion for a person to be “deemed disabled,” as is specified in the US Medicare entitlement for the purpose of covering kidney patients’ treatment costs. The key question posed was whether impairments associated with chronic kidney failure correlate with an inability to work for 12 consecutive months. However, the study team concluded that it was not possible to answer this question, in large part due to the challenge of ascertaining inability to work. The consensus was that it is only possible to infer inability to work using measures such as self-reported ability/inability to work [13].

Although work “ability” and work “inability”/“disability” are elusive concepts, feeling able to work is a logical prerequisite for employment. Job requirements and employer expectations are important influences on whether a person is able to continue working at a particular job after the start of RRT. In addition, the availability of disability benefits for ESKD patients in the US is an acknowledged influence on whether persons who consider themselves able to work do actually remain in the labor force [4,12,14]. However, with regard to feeling able to work, a key factor in sociological terms may be the balance that people experience between “good days” and “bad days”—a phenomenon common to many people who have a chronic illness [15]. Quality of dialysis care is relevant for this balance. 

### 1.3. Dialysis Care Quality Metrics

Increasing the value of healthcare requires efforts to maximize quality, lower cost, or both [16]. Meeting quality of care criteria should benefit individuals’ health and potentially reduce the incidence of costly hospitalization and medical procedures. Thus, quality incentive program (QIP) metrics have been adopted with the goal of promoting quality health care that is patient-centered as well as outcome-oriented [6]. 

In 2008, the US Medicare Improvements for Patients and Providers Act (MIPPA) legislated the linkage of payment for dialysis services to quality measures. A QIP directed to ESKD care—the first federal pay for performance program in the US—was initiated for payment year (PY) 2012. Proposed metrics for subsequent years now extend through PY 2020 [6]. For each PY, the calendar year for QIP dialysis facility data is two years prior. 

MIPPA mandated that the QIP include measures of dialysis adequacy and anemia management—aspects of dialysis care that have important implications for ESKD patient morbidity and mortality (e.g., [17]). Additional measures have been recommended to be included in the QIP “to the extent feasible” [6]. The emphasis has been on additional measures of dialysis care (vascular access type, bloodstream infection, calcium and phosphorus levels) and standardized facility ratios for transfusion and hospital readmission events (Table 1).

Patient-centered measures to assess depression, pain, and satisfaction with care experience were introduced for the PY 2018 QIP. However, a recent overview of the QIP’s evolution since PY 2012 alleged that the QIP “still has far to go” to address issues that matter most to patients. The authors observed that “the QIP has substantially expanded since introduction,” and that this “lack of parsimony threaten[s] true quality improvement activities that focus on the most important patient-centered aspects of quality care” [6]. 

In data obtained for a large prevalent HD cohort, we examined ability to work reported by working-age patients in relation to five current QIP metrics (Table 1); i.e., indicators of Kt/V dialysis adequacy, anemia management, current vascular access type, mineral metabolism, and depression. It has been common for QIP indicators to undergo minor modifications over time in how they are operationalized [6]. The dialysis adequacy, anemia management, vascular access, and mineral metabolism targets we examined were consistent with those measures’ definition at the time of our data collection. Depression had not yet been added to the QIP when our study was conducted, but participants in our study did complete one of the validated depression screening tools that are currently recommended for this purpose [18].

## 2. Materials and Methods

### 2.1. Study Population

The source of data for this investigation of work ability and QIP metrics is a multi-center study of prevalent patients on HD known as the ACTIVE-ADIPOSE Study (AAS) [19]. In conjunction with the United States Renal Data System (USRDS), we participated in coordinating and conducting the AAS at fourteen outpatient dialysis clinics located in the Atlanta, Georgia metropolitan area and the San Francisco Bay Area, California, USA. Dialysis clinics included in the study were affiliated with large dialysis providers, medium-size providers, and academic medical centers. Institutional review boards at Emory University and the University of California-San Francisco approved the study. Baseline evaluations of participants were conducted 2009–2011 and are the source of the data that we report below. 

Eligible study participants were adults (≥18 years old), English- or Spanish-speaking, on HD for at least 3 months, and capable of giving informed consent. Exclusion criteria were current treatment by peritoneal dialysis or home HD; evidence of active malignancy, including brain tumor; and expected geographic relocation. Vulnerable populations (pregnant women, prisoners, persons with significant mental illness) were also excluded. Single and double amputees and patients with prior or pending transplantation were considered eligible. Among eligible patients, 85% supplied informed consent and were enrolled. Reasons most frequently given by those who declined to participate were that they were “not interested,” “too busy,” or “enrolled in another study.” 

AAS participants were similar to the US prevalent in-center HD population in male/female distribution and in the proportion of patients with diabetes and hypertension as primary causes of ESKD. The study included higher proportions of black patients and patients of other races compared with the overall in-center HD population, as would be expected from the selected study sites, and the overall age of participants was younger. Participants’ median length of time receiving ESKD treatment was 3.3 years.

This paper focuses on the 528 study participants who were potentially working-age (i.e., ages 20–64). These participants comprised 68% of the total AAS cohort; their characteristics are summarized in Table 2.

### 2.2. Measurement

Study coordinators reviewed medical records and conducted a brief standardized interview with participants. Medical record review was the source of metrics assessing QIP target goals for dialysis adequacy (Kt/V ≥ 1.2), anemia management (hemoglobin level = 10–12 g/dL), and mineral metabolism (serum phosphorus level predialysis = 3.5–5.5 mg/dL). Laboratory values were abstracted from medical records for the date closest to the study interview date when patients reported ability to work. The patient’s current vascular access type was ascertained at the time of the interview.

As noted above, QIP indicators have had minor modifications over time in exactly how they are operationalized. In order to provide an overview of the current metrics included in the QIP, Table 1 lists the indicators that dialysis facilities must report for PY 2019 using 2017 patient data. At the time of our baseline AAS data collection, the QIP dialysis adequacy indicator was urea reduction ratio (URR) ≥ 65%, rather than Kt/V ≥ 1.2. Both of these measures are reported in medical records and are viewed as indicators of dialysis treatment adequacy; Kt/V adequacy has been the required indicator in patient data reported for 2013 to the present. The anemia management QIP indicator and the mineral metabolism QIP indicator at the time of our study did not differ from QIP indicators for those metrics that must be reported for the current year (i.e., 2017). Current (2017) vascular access type reporting requires information about patient-months with an arteriovenous fistula ≥90 days as well as patient-months with a catheter ≥90 days, reflecting efforts in the renal community to increase use of fistulas and minimize use of catheters for vascular access as much as possible. Thus, the current vascular access indicator is a combined measure rather than being based on the presence/absence of a catheter for vascular access as was previously the case [17]. In the AAS, study coordinators recorded the patients’ type of vascular access in use at the time of the patient interview. For the present study, vascular access type was categorized as catheter/no catheter in use. 

During the interview, AAS participants were asked “Are you now able to work for pay (full-time or part-time)?” and “Are you now working for pay (receiving taxable wages), full-time or part-time?” Participants also completed the Center for Epidemiologic Studies-Depression (CES-D) scale [20], which provided a measure of depressive symptomatology. Among dialysis patients, a CES-D score of 18 or higher is suggestive of clinical depression [21].

The AAS dataset can be requested from the USRDS Coordinating Center via submission of a Data Use Agreement form available on the website: www.usrds.org.

### 2.3. Analysis

Participants were classified by reported ability to work (yes/no). Patient characteristics shown in Table 2 were compared by respondents’ reported work ability, using chi-square tests for categorical variables and *t*-test or Wilcoxon test as appropriate for continuous variables. Among study participants who reported/did not report work ability, the percentage who met each of the four treatment-specific quality metrics, and the percentage with a CES-D score of 18+, were compared via chi-square test. Statistical analyses were conducted using SAS, version 9.3 (SAS Institute Inc., Cary, NC, USA). 

## 3. Results

Almost 36% (189/528) of study participants aged 20–64 reported that they were currently able to work. These patients were younger on average (46.7 vs. 52.3 years old), more likely to have at least a high school education (82% vs. 75%), and less likely to have diabetes (26% vs. 38%) than were their counterparts who said that they were not able to work. However, participants who reported (and did not report) being able to work did not differ significantly with respect to gender, race, presence of congestive heart failure, or length of time that they had been receiving ESKD treatment. 

As shown in Table 3, whether they reported they were *able* or *not able* to work, a Kt/V of 1.2 or higher was observed for 90% or more of the study cohort. Average hemoglobin in the range of 10–12 g/dL was observed for approximately 60% of each group. Serum phosphorus level in the range of 3.5–5.5 mg/dL was observed for approximately 50% of each group. However, non-use of a catheter for vascular access characterized almost 85% of those who reported ability to work, compared with about 74% of those who reported that they were not able to work (*p* = 0.004). Finally, 18.6% of participants who reported ability to work had a Center for Epidemiologic Studies-Depression (CES-D) score of 18+, while 31.6% of participants who said they were not able to work had a CES-D score of 18+ (*p* = 0.001).

## 4. Discussion

In a large cohort of working-age persons receiving center-based maintenance HD, the proportions who met QIP targets for dialysis adequacy, anemia management, and mineral metabolism were similar regardless of whether or not patients reported that they were currently able to work. However, use of a catheter for vascular access and CES-D scores that indicated depressive symptomatology were more frequent among patients who said they were not able to work. In fact, compared with patients who reported ability to work, almost twice as many patients who said they were not able to work had a CES-D score that suggested depression. 

More than 90% of the overall study cohort met the target for dialysis adequacy, which specifies that the Kt/V for a patient’s delivered dialysis dose should be 1.2 or higher [22]. Kt/V, or urea kinetic modeling, is a way to measure the amount of urea removed during HD. The proportion who attained hemoglobin and serum phosphorus targets did not differ for patients who reported (and did not report) being able to work, although smaller proportions attained these outcomes than was true for the dialysis adequacy target, which may reflect lower consensus about clinical management of these two areas. For example, a current view is that individualized hemoglobin targets should remain an option in patient care [23], and data from a large international study indicated that it is rare for dialysis patients to have values in the normal range on all indicators of mineral metabolism [24]. 

In our study cohort, fewer patients who reported ability to work used a catheter for vascular access. Morbidity, especially risk of infection and the potential for inadequate blood flow during dialysis, is increased among HD patients who use a catheter [17], and in an earlier study we showed an association between catheter use and activity limitation [25]—a factor that may have an important influence on perceived ability to work. At the same time, although arteriovenous fistulas are the preferred type of access for patients on HD, pain during needle insertion and thrombosis of the access can be problems [26]. Patients’ assessment of their dialysis access experience could be an informative quality of care metric. 

With regard to assessment of depression, the US QIP program is currently in the second year of routine screening for depression in dialysis clinics. The QIP depression reporting measure that was recently added to the QIP refers to both screening for depression and documentation of a treatment plan when the screen indicates possible depression [18]. Our data are relevant for the screening dimension. Depression is an under-recognized and undertreated issue that may characterize 25–30% of all patients on dialysis, and depressed mood may be one of the most important metrics associated with patients’ perceived ability to work. An association between ESKD patients’ depressed mood and reduced occupational activity/employment has been well documented in the literature [14,27,28,29,30]. It is of interest that in one randomized clinical trial, depressed patients who were identified in primary care practices had a higher chance of remaining employed for at least 1 year when they received treatment in a quality-improvement program that addressed depression, and this outcome included both patients with a 12-month or lifetime disorder and patients with depressive symptoms only [31]. 

Improving depressed mood could help to promote employment, and productive activity may be important for improving mood. In an earlier study, we interviewed a young man with a high school education and minimal work experience who recalled that after starting dialysis “I would just stay in the bed and go to sleep, didn’t care for myself or nothing like that…Then I got me a job. I mean it ain’t a job, just help a guy out, but clean up the shop, run errands for him, paint tires a little bit, and it would help me to think for myself and make me feel better…” [32] (p. 55).

No other studies have examined how specific quality of care indicators may be associated with dialysis patients’ reported ability to work or employment status. We previously examined the association of incremental increase in clinical targets met by working-age AAS participants with their likelihood of reporting ability to work. We found that after adjusting for patient characteristics and clustering by dialysis facility, patients’ likelihood of reporting ability to work increased with the number of clinical targets met [33,34]. In this paper, we have examined whether specific quality indicators differentiated individuals who perceived themselves as work-able as opposed to not work-able, which may suggest potentially actionable aspects of dialysis care that could be relevant for patients’ success in combining maintenance dialysis with some type of gainful employment. 

Dialysis providers’ support for patient goals regarding ability to work and employment could be considered as a QI measure. A recent policy perspective argued that the Centers for Medicare & Medicaid Services (CMS) should support activities to promote the initiation of more timely counseling about employment, as well as ESKD treatment options, for patients who are expected to require RRT [12]. The primary objective would be to facilitate ESKD patients’ maintenance of their pre-dialysis employment. These activities, directed by the regional “ESRD Networks” that oversee the Medicare program for CMS, could have important implications for patient-perceived ability to work and continued employment. Individuals who are able to continue jobs that they held prior to initiating dialysis are much more likely to remain in the labor force, and employment support should ideally begin well before a person initiates RRT. However, about 38% of individuals who start maintenance dialysis in the US do not receive pre-ESKD care [7]. Providing support for job maintenance is therefore important not only pre-ESKD, but also in the dialysis setting. 

Consistent with other studies that have considered both reported work ability and rates of actual employment among dialysis patients, fewer members of our study cohort were currently working for pay than reported being currently able to work. Many challenges can contribute to the gap between feeling able to work and actually working, including job requirements, employer expectations, and availability of disability benefits. We include a brief case study below that illustrates some of the multiple factors that may impinge on employment in addition to the clinical factors emphasized in QIP metrics.

Julia, the focus of our case study, clearly derived important psychosocial benefits from her job and believed that her contributions were valued by her colleagues. Importantly, her physician recognized her desire to continue working once she started dialysis and was aware that losing this valued role could be a precursor for depression. Her physician recommended that Julia consider home dialysis, which generally provides more flexibility for combining treatment and employment. However, she preferred going to the dialysis clinic, where she felt that she benefitted from the support she experienced while undergoing dialysis treatment with others. At the same time, her experience with vascular access problems was a serious concern—a clinical care issue requiring hospitalizations that made her question whether continuing her job was realistic.

**African-American Female Age 54 on In-center Hemodialysis** Soon after she began dialysis, she “was thinking about retiring” because of the problems she was having with her vascular access. She “never knew when I would be in the hospital.” However, she “liked her job and didn’t want to stop working.” In fact, she “never thought much about not working. Only when I was being in the hospital so much, but it wasn’t because I didn’t want to work.” She was concerned about the work that was not getting done because of her frequent, unscheduled absences. But her doctor encouraged her not to quit. He said, “Go part-time if you feel too bad. It will keep you from getting depressed.” If she had worked in a stressful situation, she feels it would have made a difference, but her work atmosphere is not stressful. In fact, she said “they work around me.” She didn’t want to use her sick leave to go to dialysis. She leaves work early on Wednesdays so that she can dialyze and still go to her Wednesday evening church activities. Then she works one hour extra on Tuesdays and Thursdays to make up for leaving early on Wednesdays. On Mondays and Fridays, she works a full day before leaving for treatment. “So I don’t have to use leave.” Her doctor had initially wanted her on home dialysis, but she told him “that won’t work for me. I like being around people.” She enjoys the social aspects of her job and of the dialysis clinic: “I need a support group!”** Summary of Study Participant Interview Comments (NIH grant R01-DK42949: Gender, Race, Age and ESRD Patients’ Quality of Life and Survival)**

## 5. Conclusions

Quality improvement activities for dialysis care have been a prominent focus of the US renal community for a number of years, pre-dating the initiation of the QIP [35]. These activities have emphasized indicators of clinical performance in the delivery of dialysis care. Currently, the importance of including more patient-centered aspects of quality care is receiving growing attention [6,12]. A QIP measure(s) that addressed support for patients’ employment goals could be applicable for more than half of the patients currently receiving center-based HD in the US (i.e., those in the potential working-age range), with implications for reducing the personal and societal economic burden of ESKD [36]. Moreover, a latent function of patient-centered metrics may be increased motivation for provider awareness and provider–patient communication about patients’ feelings and priorities, which could have important benefits apart from the implementation of specific interventions. Dialysis quality assessment is increasingly challenged to identify key clinical performance targets that are essential to monitor, while also addressing patient preferences across age and care continua to support patients’ priorities for their lives, including opportunities for productive activity.

## Figures and Tables

**Table 1:** QIP measures payment year (PY) 2019 (Calendar year for patient data is 2017).

**Clinical Measures**	Kt/V adequacy
	Vascular access type
	Hypercalcemia (calcium level)
	Dialysis-related bloodstream infections
	Standardized transfusion ratio
	Standardized readmission ratio: readmissions within 30 days of an index discharge
	Patient experience survey (ICH CAHPS)
**Reporting Measures**	Anemia: patient-specific hemoglobin + monthly ESA dosage (as applicable)
	Mineral metabolism (serum phosphorus level)
	Depression
	Pain
	Healthcare personnel influenza vaccination

*Abbreviations*: ESA, erythropoietin stimulating agent; ICH CAHPS, In-Center Hemodialysis Consumer Assessment of Healthcare Providers and Systems; QIP, Quality Incentive Program. *Note*: Additional measures for PY 2020 are ultrafiltration rate and standardized hospitalization ratio.

**Table 2:** Characteristics of HD patients aged 20–64 in the USRDS AAS at the time of baseline evaluations 2009–2011.

Characteristics	Working-Age Study Cohort (*n* = 528)
Age in years, mean (S.D.)	50.3 (10.4)
Male, %	62.3
Black, %	66.5
At least high school education, %	77.7
Diabetes, %	34.1
Congestive heart failure, %	16.3
ESKD treatment in years, median (range)	3.4 (0.1, 36.6)

*Abbreviations*: AAS, ACTIVE-ADIPOSE Study; ESKD, end-stage kidney disease; HD, hemodialysis; USRDS: United States Renal Data System.

**Table 3:** Attainment of quality indicator goals: working-age ESKD patients on HD who reported ability to work and did not report ability to work.

QIP Targets	Ability to Work Reported Yes (*n* = 189) No (*n* = 339)		*p*
Kt/V ≥ 1.2, %	92.1	90.0	NS
Hemoglobin 10–12 g/dL, %	60.9	59.0	NS
Serum phosphorus mg/dL, %	48.2	50.2	NS
No catheter in use for vascular access, %	84.7	73.8	0.004
CES-D score ≥ 18, %	18.6	31.6	0.001

*Abbreviations*: CES-D, Center for Epidemiologic Studies-Depression; ESKD, end stage kidney disease; HD, hemodialysis.
